# Who does not LYKe fungi? A plant receptor modulates defenses to facilitate the establishment of fungal symbioses

**DOI:** 10.1093/plphys/kiad134

**Published:** 2023-02-28

**Authors:** Manuel González-Fuente

**Affiliations:** Assistant Features Editor, Plant Physiology, American Society of Plant Biologists, USA; Faculty of Biology and Biotechnology, Ruhr-University Bochum, Bochum, Germany

Root symbioses allow plants to increase their effective root surface and improve the uptake of nutrients and water. Arbuscular mycorrhizae, a type of symbiosis between plant roots and certain fungi, trace back almost as far as the adaption of plants to land and are present in up to 80% of plant species (Brundrett and Tedersoo 2018). It is thought that the ability to establish these symbioses was the ancestral condition that was later lost in some species.

To establish these symbioses, arbuscular mycorrhizal fungi (AMF) secrete signaling molecules called lipo-chitooligosaccharides (LCOs). LCOs are recognized by plant receptors to activate the signaling pathway required for the establishment of the symbiosis (Venkateshwaran et al. 2013). However, as with any other fungi, AMF also present highly conserved molecules that are perceived by the plant innate immune system and trigger broad-spectrum defense responses against pathogens (Guo and Cheng 2022). To allow the colonization of symbionts and prevent the invasion of pathogens, plants need to fine-tune their defense responses.

Most of what we know today about the modulation of defenses upon perception of symbionts comes from legumes (Benezech et al. 2020). Most legumes form a different type of symbiosis with nitrogen-fixing bacteria that also produce LCOs that are recognized by the plant to activate the symbiotic pathway and interfere with plant defenses (Rey et al. 2019). However, how other plant families modulate their defenses to accommodate symbionts has still not been fully understood.

**Figure 1.**
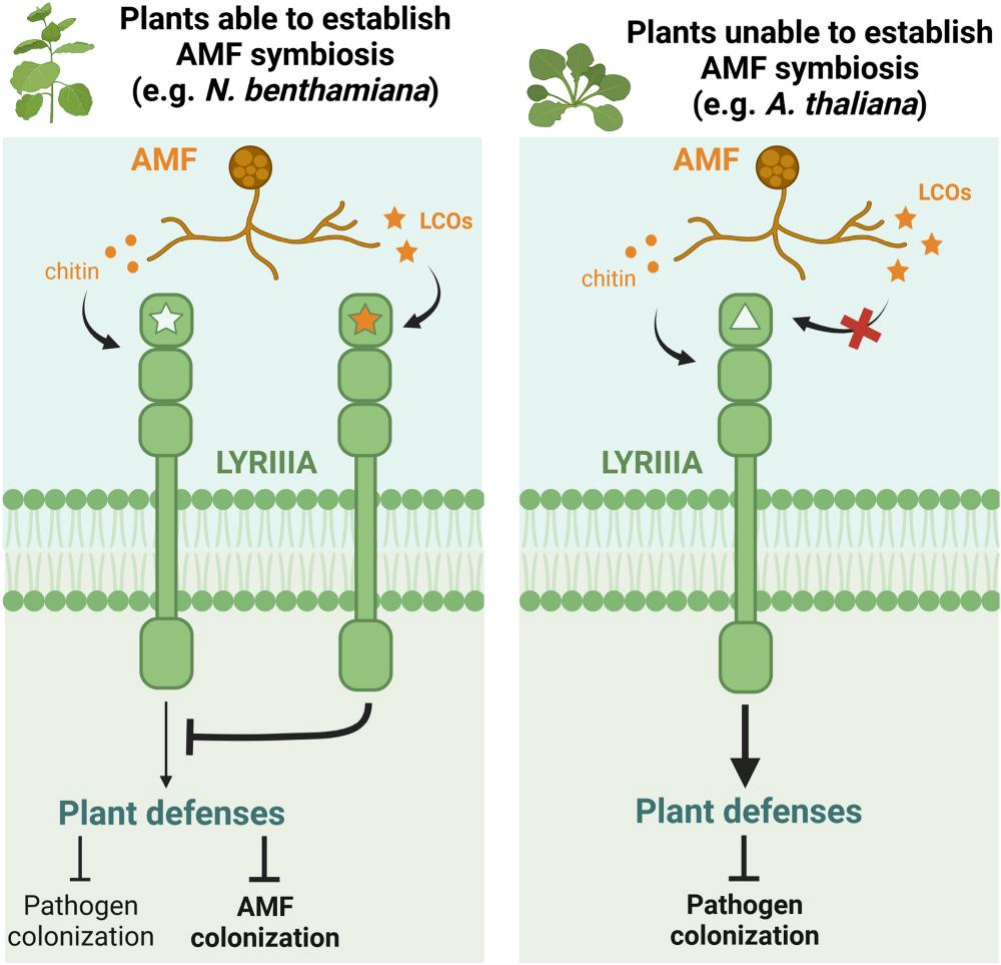
LYRIIIA receptors modulate immunity upon LCO perception to facilitate AMF colonization in plants able to establish symbioses. LYRIIIA (LYK-RELATED IIIA) receptors are involved in mounting defense responses triggered by conserved fungal molecules such as chitin. LYRIIIA receptors from plants able to establish AMF symbiosis, such as *N. benthamiana* (left), recognize the LCOs secreted by AMF to compromise the plant defense responses and subsequently facilitate AMF colonization. However, the LYRIIIA receptors from plants that have lost the ability to establish symbiosis, such as *A. thaliana* (right), are unable to bind LCOs and therefore only maintain their ability to positively regulate plant defenses. Illustration based on Wang et al. (2023), created with BioRender.com.

In this issue of *Plant Physiology*, Wang et al. (2023) explored the function of LCOs, specifically their roles in suppressing defenses and promoting symbiosis, in the nonlegume *Nicotiana benthamiana*. The authors reported that upon perception of LCOs in *N. benthamiana*, a conserved receptor modulates plant defenses to facilitate AMF colonization (Fig. 1). This protein, called NbLYK4 (LYSM-CONTAINING RECEPTOR-LIKE KINASE 4), belongs to the LYRIIIA (LYK-RELATED IIIA) family of LCO-binding receptors present in all land plants (Buendia et al. 2018).

Previous studies have shown that LYRIIIA receptors are involved in defenses against pathogens in legumes and Arabidopsis (*Arabidopsis thaliana*) (Wan et al. 2012; Fuechtbauer et al. 2018). In the present study, the authors demonstrated that NbLYK4 is involved in plant defenses in a solanaceous species. They showed that *NbLYK4*-silenced plants are more susceptible to infection with various pathogens because they are unable to mount appropriate defense responses. Moreover, when introduced in the corresponding Arabidopsis *atlyk4* mutant, NbLYK4 complemented the defect in defense responses of the mutant. These findings suggest that LYRIIIA receptors have a conserved role in modulating plant defenses, including in species unable to form root symbioses such as Arabidopsis.

Previous studies in legumes have shown that LYRIIIA receptors from species that are unable to establish mycorrhizal symbiosis (e.g. certain *Lupinus* species) are also unable to bind LCOs with high affinity (Malkov et al. 2016). To test whether this association also applies to nonlegume plants, Wang et al. (2023) compared the LCO-binding properties of NbLYK4 and its peach (*Prunus persica*) and Arabidopsis orthologs. Both *N. benthamiana* and peach form symbioses with AMF, whereas Arabidopsis does not. Indeed, the authors observed that the receptors from the symbiosis-forming species *N. benthamiana* and peach bind LCOs, whereas the receptor from the nonsymbiosis-forming Arabidopsis does not. This expands the association between LCO-binding and the ability of the plant to establish root symbioses to nonlegume plants.

LCO perception inhibits plant defenses in legumes (Rey et al. 2019). In the present study, the authors showed that LCO perception also compromises plant defense responses in solanaceous *N. benthamiana*. Moreover, they observed that LCO-mediated inhibition of defenses did not occur in *NbLYK4*-silenced plants. This suggests that NbLYK4 is the LCO receptor responsible for the LCO-mediated inhibition of defenses.

Next, the authors tested whether NbLYK4 has a role in AMF colonization. They observed that *NbLYK4* expression was induced upon infection with AMF and that *NbLYK4*-silenced plants had higher rates of AMF colonization, consistent with the reduced defense responses observed in *NbLYK4*-silenced plants. Altogether, these findings suggest that NbLYK4-mediated modulation of plant defenses allows AMF to penetrate and spread faster in the plant.

In summary, Wang et al. (2023) showed that LCO-mediated modulation of plant defenses to facilitate symbioses also occurs in nonlegumes that are able to establish arbuscular mycorrhizae. Moreover, the presence of LYRIIIA receptors in all plants, including species such as Arabidopsis that are unable to establish root symbioses, favors a scenario in which the LYRIIIA ancestor was probably able to bind LCOs and modulate defenses in ancient land plants. While beneficial for the establishment of root symbioses, this LCO-mediated modulation of defenses also constitutes a vulnerability that could be potentially exploited by pathogenic fungi able to secrete LCOs. Indeed, LCOs are produced by a wide variety of fungi, not exclusively symbionts (Rush et al. 2020), which would explain why LYRIIIA receptors from plants that have lost the ability to establish root symbioses have lost their biochemical ability to bind LCOs, maintaining only their positive effect on defenses. This scenario would constitute an elegant example of adaptive counterselection in Arabidopsis and possibly other plant species that do not form symbioses.

In the long term, this work will contribute to a better understanding of the molecular mechanisms ruling the tradeoff between immunity and symbiosis that has traditionally been mostly studied in legume/bacterial symbioses (Benezech et al. 2020). Wang et al. (2023) demonstrate that similar mechanisms also apply to nonlegume species such as *N. benthamiana*. Understanding these processes will be crucial in the future to generate new crop varieties that establish symbioses more easily and/or efficiently, without compromising their resistance to pests and diseases in the process, and could allow us to reduce the application of fertilizers and pesticides, bringing us a step closer toward a more productive and sustainable agriculture.
